# Effect of Pterygomasseteric sling shortening via mandibular angle resection on postoperative skeletal stability in skeletal class III malocclusions treated with mandibular setback surgery

**DOI:** 10.1016/j.jpra.2026.05.035

**Published:** 2026-05-23

**Authors:** Le Tan Hung, Pham Trinh Quoc Khanh, Le Duc Lanh, Tran Ai Khiem, Nguyen My Huyen, Vo Ke Dat

**Affiliations:** a Faculty of Odonto – Stomatology, College of Medicine and Pharmacy of Tra Vinh University, Vinh Long Province, Vietnam; b Can Tho University of Medicine and Pharmacy, Can Tho City, Vietnam; c Faculty of Odonto – Stomatology, Hong Bang International University, Ho Chi Minh City, Vietnam; d Trung Vuong Hospital, Ho Chi Minh City, Vietnam

**Keywords:** Orthognatic surgery, Mandibular setback, Skeletal class III maloccusion, Skeletal stability

## Abstract

Postoperative skeletal stability issues are frequently attributed to the tension exerted by the pterygomasseteric muscle sling following mandibular setback. This study investigated the hypothesis that simultaneous mandibular angle resection during Bilateral Sagittal Split Ramus Osteotomy (BSSRO) enhances skeletal stability by reducing this muscular tension.

A prospective randomised controlled study was conducted on 20 patients with Skeletal Class III malocclusion - characterised by mandibular prognathism. The study sample was equally divided into an experimental group *(4 males, 6 females; mean age 23.6±3.9 years)* undergoing the combined procedure (BSSRO with simultaneous mandibular angle ostectomy), and a control group *(5 males, 5 females; mean age 20.4±3.4 years)* receiving isolated ramus osteotomy (conventional BSSRO setback only). Lateral cephalometric tracings were utilised to quantify the horizontal and vertical positional changes of the mental region, specifically at Point B, Pogonion and Menton, during the 3-month postsurgical healing phase.

The results indicated a significant divergence in remodelling between the two groups. The control group characterised anterior relapse (B: +1.35 ± 1.50 mm; Pog: +0.81 ± 2.92 mm), whereas the experimental group demonstrated a stabilising posterior settling effect (Point B: -1.44 ± 1.93 mm; Pog: -1.14 ± 4.06 mm).

These findings substantiate that resecting the mandibular angle effectively shortens the pterygomasseteric sling, thereby alleviating the muscular tension responsible for skeletal rebound. Consequently, simultaneous angle ostectomy during Surgery-First Orthognathic Approach (SFOA) mandibular setback protocol is advocated not merely as an aesthetic contouring procedure for lower facial width reduction, but as a functional release of the pterygomasseteric sling that significantly improves early skeletal stability, suggesting a larger study with Class II malocclusions as well or other types of skeletal deformity

## Introduction

In contemporary craniomaxillofacial surgery, the treatment paradigm for mandibular prognathism has evolved from purely functional occlusal correction to a comprehensive approach to enhancing facial harmony.^1^, ^2^, ^3^ While Bilateral Sagittal Split Ramus Osteotomy (BSSRO) remains the gold standard for mandibular setback, it presents a distinct aesthetic paradox, particularly in Asian populations where a slender, oval facial contour is highly desired.^4^ The posterior repositioning of the distal segment during BSSRO often exacerbates the prominence of the mandibular angle or leads to an increase in intergonial width, resulting in a coarse, quadrangular lower facial appearance rather than the desired tapered profile.^5^^,^^6^

Additionally, the Surgery-First Orthognathic Approach (SFOA) has emerged as a paradigm shift in contemporary orthognathic surgery, offering distinct advantages, including reduced total treatment time and immediate resolution of facial esthetics by eliminating the presurgical decompensation phase.^4^^,^^7^ Despite the benefits of SFOA, postoperative skeletal stability remains a critical challenge, as the absence of rigid intercuspation renders the osteotomized segments highly susceptible to relapse forces exerted by the pterygomasseteric muscle complex.^8^, ^9^, ^10^, ^11^, ^12^, ^13^ The aetiology of relapse is multifactorial, involving condylar positioning, fixation methods, and complex neuromuscular adaptations.^14^ A dominant factor governing this instability is the tension generated within the pterygomasseteric muscle complex. During setback surgery, the proximal segment may undergo rotational changes that disrupt the physiological length of the pterygomasseteric sling.^15^, ^16^, ^17^ Recent advances in 3D-guided surgery have demonstrated the feasibility and safety of simultaneous protocols; however, the biomechanical impact of this combined approach on skeletal retention remains unclear.^10^^,^^18^^,^^19^ Current literature suggests that if the proximal segment is inadvertently rotated or the muscle sling remains stretched, the resultant tension exerts an anterior vector force during the healing phase, predisposing the mandible to significant skeletal relapse.^14^^,^^15^ Therefore, simultaneous mandibular angle resection is increasingly advocated not merely as an esthetic adjunct, but as a strategic biomechanical intervention to neutralise this muscular tension and optimise long-term skeletal retention.

It is hypothesised that performing mandibular angle resection concomitantly with BSSRO offers a synergistic benefit: it not only refines the transverse facial dimension for superior aesthetics but also enhances stability by shortening the attachment site of the pterygomasseteric sling. Therefore, the main objective of this study was to evaluate the efficacy of simultaneous mandibular angle resection and BSSRO in reducing early postoperative skeletal instability compared to isolated BSSR. In the context of this study, 'skeletal stability' is specifically defined as the resistance to early postoperative muscular traction, quantified by the degree of osseous relapse from the immediate postoperative baseline (T1) to the end of the consolidation phase (T2). The null hypothesis was that simultaneous mandibular angle resection during BSSRO does not significantly affect early postoperative skeletal stability compared to isolated BSSRO.

## Materials and methods

***Study Sample:*** A prospective randomised controlled study was conducted of 20 patients diagnosed with skeletal Class III malocclusion secondary to mandibular prognathism, treated at the Department of Plastic Surgery, Van Hanh General Hospital, Ho Chi Minh, Viet Nam (IEC. Ref number 01/2025/GCN-HĐĐĐ)

All participants underwent BSSRO via surgery-first orthognathic approach (SFOA) surgery. This protocol was selected to leverage the regional acceleratory phenomenon (RAP) for faster postoperative tooth movement.^20^ Strict exclusion criteria were applied to isolate the biomechanical effects of mandibular setback: patients requiring concurrent maxillary Le Fort I osteotomy or genioplasty, or those presenting with craniofacial syndromes, were excluded. Consequently, the study focused exclusively on isolated mandibular setback procedures. The study population was allocated into two parallel groups using a simple randomisation method (computer-generated random numbers) by a research assistant not involved in the surgical procedures. The experimental group (*n* = 10) underwent bilateral sagittal split ramus osteotomy (BSSRO) with simultaneous mandibular angle ostectomy, while the control group (*n* = 10) received conventional BSSRO setback only.

***Surgical Interventions*:** A fully digital workflow was implemented, including preoperative high-resolution cone-beam computed tomography (CBCT) scans acquired for all subjects. The DICOM data was imported into advanced medical image processing software (Mimics, Materialise, Leuven, Belgium) to reconstruct three-dimensional (3D) virtual skull models. Both groups utilised computer-aided design and manufacturing (CAD/CAM) technology to fabricate 3D-printed surgical guide templates, which were applied intraoperatively, based on virtual simulation, to precisely direct the ostectomy line below the inferior alveolar nerve canal while ensuring bilateral symmetry of the mandibular lower border. As hypothesised in previous stability studies, the treatments reduce pterygomasseteric sling tension.^15^ To eliminate performance bias and ensure strict technical homogeneity, all surgical procedures across both the experimental and control groups were performed by a single, highly experienced maxillofacial surgeon and the same dedicated surgical and postoperative care team. The ostectomy protocol, guided by the 3D-printed templates, was strictly standardised for all patients. Rigid internal fixation (RIF) using miniplates and monocortical screws was applied in all cases to secure the proximal and distal segments. For the experimental group, following the bilateral sagittal split ramus osteotomy (BSSRO), the prominent mandibular angle was resected under direct visualisation using a reciprocating saw, guided by a prefabricated template to ensure precise contouring (Fig. 1). A distinct advantage of this simultaneous protocol is the immediate biomechanical shortening of the pterygomasseteric sling, which effectively decompresses the muscular tension responsible for postoperative skeletal relapse.^15^ Postoperative orthodontic treatment commenced following the resolution of acute inflammation to settle the occlusion.^20^

**Fig. 1:**
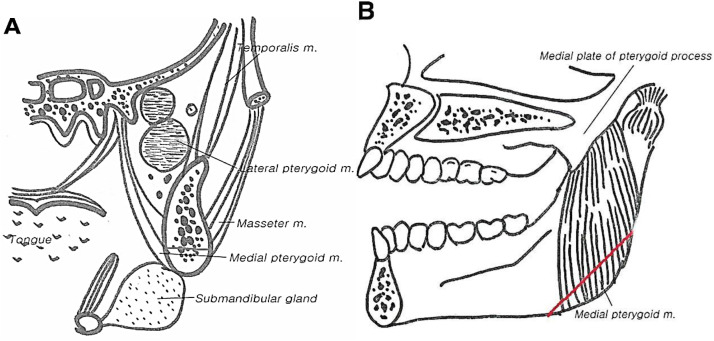
Pterygomasseteric muscle sling shortening by mandibular angle resection. Frontal (A) and sagittal (B) views.^15^

***Radiographical assessment and variables recorded:*** Serial lateral cephalograms were acquired at three distinct intervals to facilitate direct comparison with the established literature by Kim et al. (2007): preoperative (T0), immediate postoperative (T1), and three-month follow-up (T2). To minimise measurement bias and ensure rigorous blinding, all postoperative lateral cephalograms were coded, stripping patient identifiers and group allocations. A single, independent orthodontist, who was strictly blinded to the surgical interventions, patient identities and study objectives, performed all cephalometric tracings and digitisations. To establish intra-examiner reliability, 20% of the total cephalograms (*n* = 12) were randomly selected and re-traced by the same examiner after a two-week washout period. The intra-class correlation coefficient (ICC) was calculated using a two-way mixed-effects model with absolute agreement. Measurements of Point B, Pogonion, Menton, and the SNB angle were subjected to this reliability analysis. A coordinate system anchored to the Sella-Nasion (SN) reference plane was established to evaluate the horizontal (X) and vertical (Y) displacements of the mandibular symphysis landmarks: Point B, Pogonion (Pog), and Menton (Me) (Fig. 2).^15^ The magnitude of surgical setback was defined by the positional shift of Point B from T0 to T1. Additionally, the SNB angle was recorded as a supplemental angular metric. Descriptive statistical analysis assessed the mean changes and standard deviations of these parameters between the experimental and control cohorts at the specified time points.

**Fig. 2:**
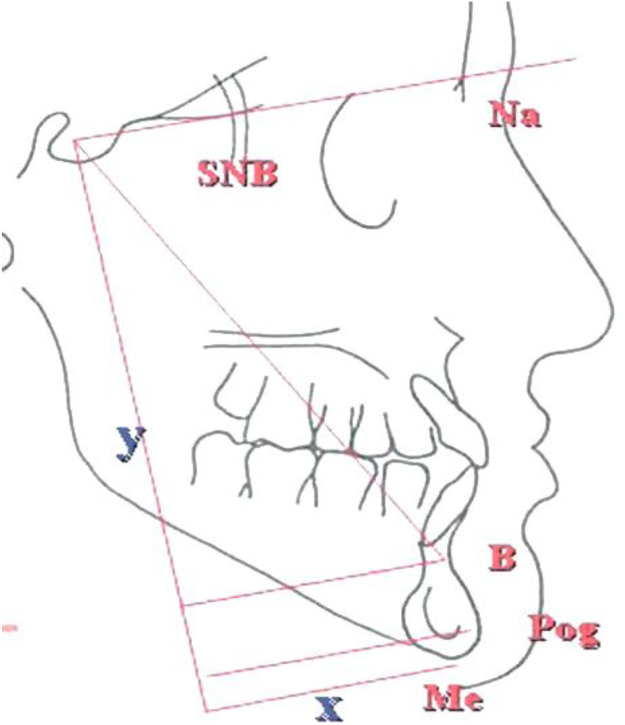
Used cephalometric tracing points to evaluate the vertical, horizontal, and angular changes by Kim et al. (2007) .^15^

***Statistical Analysis:*** Statistical analysis was performed using SPSS version 26.0 (IBM Corp, Armonk, NY). Descriptive statistics (mean and standard deviation) were calculated for all variables. Given the small sample size *(n =* *10 per group)*, nonparametric tests were employed to ensure robust statistical inference. The Mann-Whitney U test was used to compare continuous baseline variables (age, magnitude of setback, preoperative SNB) and changes in skeletal stability *(Δx, Δy)* between the two groups. Fisher’s exact test was used for the categorical variable (gender). A *p-value < 0.05* was considered statistically significant.

## Results

The intra-class correlation coefficient (ICC) analysis demonstrated excellent intra-examiner reliability for all cephalometric measurements. The ICC values ranged from 0.89 to 0.92 (95% CI: 0.80 – 0.98) for horizontal and vertical linear measurements of Point B, Pogonion, Menton, and the SNB angle. These results confirm a high degree of reproducibility, indicating that the measurement error was clinically negligible and did not confound the observed skeletal changes between the groups.

The baseline demographics and surgical parameters were evaluated to ensure comparability between the cohorts (Table 1). The mean age was 23.6 ± 3.9 years in the experimental group and 20.4 ± 3.4 years in the control group. Regarding the surgical magnitude, the experimental group achieved a mean setback of 9.28 ± 1.08 mm (with an SNB reduction of −2.37 ± 2.14°), while the control group recorded a mean setback of 8.25 ± 1.02 mm (SNB reduction of −1.09 ± 1.74°). Statistical analysis using the Mann-Whitney U test revealed no significant differences between the two groups for age (*p* = 0.063) or setback magnitude (*p* = 0.226). Furthermore, gender distribution was perfectly comparable (Fisher’s exact test, *p* = 1.000). These findings confirm that the two groups were statistically well-matched prior to the intervention, effectively minimizing baseline confounding factors (Tables 2 and 3).

**Table 1:** Baseline demographics and preoperative characteristics of the two groups.

Variables	Experimental Group (*n* = 10)	Control Group (*n* = 10)	p-value*
Age (years)	23.6 ± 3.9	20.4 ± 3.4	0.063
Gender (Male/Female)	4/6	5 / 5	1.000⁎⁎
SNB (0)	85.3 ± 1.2	84.8 ± 0.9	0.449
Surgical Setback (mm)	9.28 ± 1.08	8.25 ± 1.02	0.226

⁎ *Calculated using the Mann-*Whitney U test (p-value < 0.05).

⁎⁎ *Calculated using Fisher's exact test (p-value < 0.05).*

**Table 2:** Changes of mandible position following BSSRO and mandibular angle resection in the experimental group.

Case No.	SFOA Setback (mm)	ΔSNB (°)	B (mm)		Pog (mm)		Me (mm)	
			Δx	Δy	Δx	Δy	Δx	Δy
1	−7.5	0.45	−1.9	−2.2	−3.2	−0.8	−3.2	−2.8
2	−8.6	1.10	−0.9	1.0	0	0	0	−1.2
3	−9.8	−1.07	−4.0	−2.9	−2.8	−4.2	2.5	−3.9
4	−13.5	−0.78	−3.7	−0.5	2.4	0	1.0	−2.3
5	−8.4	−1.00	−6.8	0.5	−9.1	0	7.5	0
6	−7.0	−0.27	1.0	−3.2	0	−1.3	0	−1.5
7	−12.5	−0.15	−0.5	−1.0	0	0	−3.1	−1.0
8	−8.9	1.01	−2.3	−1.8	−2.5	−2.7	−2.0	−2.8
9	−8.2	−0.41	5.2	−2.1	3.6	0.8	3.0	−2.6
10	−11.4	−1.25	−0.5	*0*	0.2	0	−1.2	1
Mean	−9.28	−2.37	−1.44	−1.22	−1.14	−0.82	−0.45	−1.71
SD	1.08	2.14	1.93	1.46	4.06	1.73	3.72	1.73

Abbreviations: SD, standard deviation; S, Sella; N, Nasion; A, point A; B, point B; Pog, pogonion; Me, menton.

**Table 3:** Changes of mandible position in the BSSRO only group.

Case No.	SFOA Setback (mm)	ΔSNB (°)	B (mm)		Pog (mm)		Me (mm)	
			Δx	Δy	Δx	Δy	Δx	Δy
1	−9.7	1.5	−1.2	3.2	−1.3	2.0	−2.6	1.5
2	−7.5	−1.7	4.6	0	5.6	−1.7	3.5	0
3	−9.2	−1.9	0	−1.3	−1.9	−3.6	0	−3.0
4	−8.0	−0.4	−0.7	−8.1	3.2	0.5	−1.0	1.3
5	−10.5	−2.7	2.2	−4.0	2.5	−2.8	−4.1	−3.0
6	−6.5	0.2	1.5	−0.8	0	−1.5	0	−2.1
7	−8.9	−2.1	3.6	−2.2	1.8	−0.8	0	−2.6
8	−7.7	0	0	−1.0	−3.6	0.2	0	−1.0
9	−8.4	−1.8	1.0	−2.1	1.3	−3.2	−5.1	−3.6
10	−6.1	−2.3	2.5	*−5.3*	0.5	0.6	1.7	−2.6
Mean	−8.25	−1.09	1.35	−2.16	0.81	1.03	−0.76	−1.51
SD	1.02	1.74	1.50	2.72	2.92	0.56	2.09	1.88

Abbreviations: SD, standard deviation; S, Sella; N, Nasion; A, point A; B, point B; Pog, pogonion; Me, menton.

Cephalometric analysis during the consolidation phase revealed a distinct divergence in skeletal remodelling between the two protocols, particularly in the horizontal dimension. The control group (isolated BSSRO) displayed characteristic anterior relapses consistent with muscle tension forces, moving +1.35 ± 1.50 mm and +0.81 ± 2.92 mm anteriorly at Point B and Pogonion (Pog), respectively. These values indicate a partial reversion toward the preoperative Class III profile.

Conversely, the experimental group underwent concomitant angle resection demonstrated a stabilising "posterior settling" effect. Point B was displaced posteriorly by −1.44 ± 1.93 mm, and Pogonion retracted by −1.14 ± 4.06 mm. Crucially, inferential statistical analysis confirmed that this biomechanical divergence in horizontal stability at Point B was statistically significant (*p* = 0.023). Although the horizontal change at Pogonion followed a similar posterior trend in the experimental group, the difference did not reach statistical significance (*p* = 0.225), likely due to its anatomical distance from the pterygomasseteric sling insertion and the small sample size. Vertical measurements showed greater downward displacement overall, with Point B moving −1.22 ± 1.46 mm in the experimental group and −2.16 ± 2.72 mm in the control group. Similarly, Menton (Me) exhibited vertical changes of −1.71 ± 1.73 mm and −1.51 ± 1.88 mm, respectively (Table 4).

**Table 4:** Comparative differences between the experimental and the control group.

Variables		Experimental Group (Mean ± SD)	Control Group (Mean ± SD)	p-value*
*Δ SNB (°)*		−2.37 ± 2.14	−1.09 ± 1.74	*0.121*
*B*	*Δx (mm)*	−1.44 ± 1.93	1.35 ± 1.50	*0.023*
	*Δy (mm)*	−1.22 ± 1.46	−2.16 ± 2.72	*0.472*
*Pog*	*Δx (mm)*	−1.14 ± 4.06	0.81 2.92	*0.225*
	*Δy (mm)*	−0.91 ± 1.73	1.03 ± 0.56	*0.879*
*Me*	*Δx (mm)*	−0.45 ± 3.72	−0.76 ± 2.09	*0.161*
	*Δy (mm)*	−1.71 ± 1.73	−1.51 ± 1.88	*0.118*

⁎ Mann-Whitney U test, statistical significance (*p* < 0.05).

## Discussion

Based on the significant improvement in early horizontal skeletal stability at the mandibular base, the null hypothesis was rejected. The findings demonstrated a statistically significant divergence in horizontal displacement (Δx) at Point B, which acts as the most direct indicator of muscular decompression due to its proximity to the pterygomasseteric sling insertion. Although changes at the extreme anterior landmarks (Pogonion and Menton) followed a similar stabilising trend, they did not reach statistical significance. This discrepancy is biomechanically expected, as positional changes at Pogonion and Menton are attenuated by secondary confounding factors; specifically, their greater distance from the centre of rotation geometrically magnifies any slight segmental rotations, and the anterior chin region is highly susceptible to localised surface bone remodelling driven by the mentalis muscle during the consolidation phase.^28^^,^^29^

Kim et al. (2007) previously identified the pterygomasseteric sling as a primary vector for relapse, noting that inadvertent clockwise rotation or stretching of the proximal segment loads the masticatory muscles, causing anterior rebound.^10^^,^^15^ However, their findings were based on a conventional orthodontics-first protocol where solid intercuspation provides immediate mechanical resistance to muscle pull. In our study, utilising SFOA, the absence of preoperative decompensation renders the distal segment more susceptible to muscular forces.^21^ The anterior relapse observed in our control group (Table 4) aligns with findings that SFOA patients are prone to early skeletal shifting due to unstable occlusal contacts.^17^^,^^22^, ^23^, ^24^, ^25^ By performing simultaneous angle resection, we effectively partially detached the insertion of the masseter and medial pterygoid muscles.^10^^,^^12^^,^^20^ This "biomechanical decompression" neutralised the anterior vector force, allowing the mandible to settle posteriorly rather than rebound anteriorly, a phenomenon similarly observed in recent 3D analysis studies.^21^ If the proximal segment is rotated inadvertently in a clockwise direction intraoperatively, this violates the physiological harmony of the pterygomasseteric sling and results in the loading of the muscles of mastication.^11^^,^^23^^,^^26^

A notable contrast between our findings and the earlier work of Kim et al. (2007) lies in the interpretation of condylar positioning. Kim et al. cited manual condylar positioning as a confounding variable, postulating that the anterosuperior traction required for angle resection might displace the condyle, leading to pseudo-relapse.^10^^,^^15^ The consensus from recent research and a systematic review by Haas Junior OL et al. (2019) mentioned that rigid internal fixation combined with precise proximal segment management, aided here by 3D guidance, significantly enhances sagittal stability compared to manual methods.^8^^,^^10^^,^^27^ The confounding errors of manual positioning identified by Kim et al. were eliminated in our study through 3D computer-aided simulation. Instead, this posterior displacement represented a true physiological "settling" facilitated by the elimination of muscular interference, as evidenced by −1.44 ± 1.93 mm at B and −1.14 ± 4.06 mm at Pogonion, compared with anterior movement in the control group (Table 4).

The divergence in p-values across different anatomical landmarks and planes offers further insight into the biomechanical impact of angle resection. In this study, the horizontal displacement *(Δx)* at Point B was statistically significant, whereas changes at Pogonion and Menton were not. This discrepancy is clinically logical, as Point B is anatomically situated in closer proximity to the mandibular ramus and the primary insertion of the pterygomasseteric sling. Consequently, Point B acts as a more sensitive indicator of the direct 'decompression' effect following muscle detachment. The lack of statistical significance at Pogonion and Menton likely reflects the increasing influence of secondary factors, such as individual variations in symphyseal morphology and lower border remodelling, which may mask the primary muscle-driven vector. Furthermore, the non-significant results in the vertical plane *(Δy)* for all landmarks suggest that while angle resection effectively neutralises the anterior relapse vector, it does not significantly alter the vertical skeletal settling inherent to the SFOA protocol. These findings underscore that the value of simultaneous angle resection is highly specific to sagittal stabilisation, addressing the most critical relapse vector in mandibular setback surgery.

Furthermore, a distinct characteristic of SFOA is the deliberate lack of presurgical occlusal preparation, capitalizing on the Regional Acceleratory Phenomenon (RAP) for rapid postoperative bone remodelling.^20^ While the exact timing and mechanics of postoperative orthodontic intervention during the T1-T2 interval can vary with individual patient compliance, this inherent variability underscores the critical importance of primary surgical stability. By effectively shortening the pterygomasseteric sling, simultaneous angle resection acts as a fundamental biomechanical stabiliser, protecting the skeletal setback regardless of the unpredictable onset or magnitude of subsequent orthodontic forces. The discrepancy in horizontal remodelling highlights the synergy between transverse reduction and sagittal stability. Bi et al. (2025)^21^ recently demonstrated that BSSRO naturally induces lateral displacement of the proximal segment, increasing intergonial width and potentially stretching the pterygomasseteric sling laterally. In our control group, this lateral stretch likely contributed to the anterior relapse vector. In the experimental group, angle resection directly addressed this transverse dimension. By removing the bony interference and shortening the muscle sling, the procedure prevented the "flaring" effect described by Bi et al., thereby removing a secondary source of relapse tension.

In patients with small gonial angles or those requiring significant setbacks, conventional BSSRO can inadvertently create a quadrangular facial contour.^15^ Furthermore, asymmetry often persists due to differences in ramus height and visibility of the gonial angle.^17^ While Kim et al. (2007) reported a 1.04 mm anterior relapse in conventional controls,^15^ our Surgery-First control group exhibited a more pronounced relapse of 1.35 mm. Yet simultaneous resection achieved a posterior settling of −1.44 mm, compared with −1.86 mm in the Kim et al. (2007) study. This divergence indicates that the 'floating occlusion' inherent to the Surgery-First Approach amplifies muscle-driven relapse, thereby elevating the biomechanical release provided by angle resection from a beneficial modification to a functional necessity for stability. Given the higher susceptibility of SFOA cases to muscle-driven relapse, simultaneous angle resection should be considered a functional necessity rather than a cosmetic adjunct. It provides a dual benefit: immediate correction of the "squared face" morphology and a crucial enhancement of skeletal stability during the vulnerable early healing phase of SFOA (Figs. 3 and 4).

**Fig. 3:**
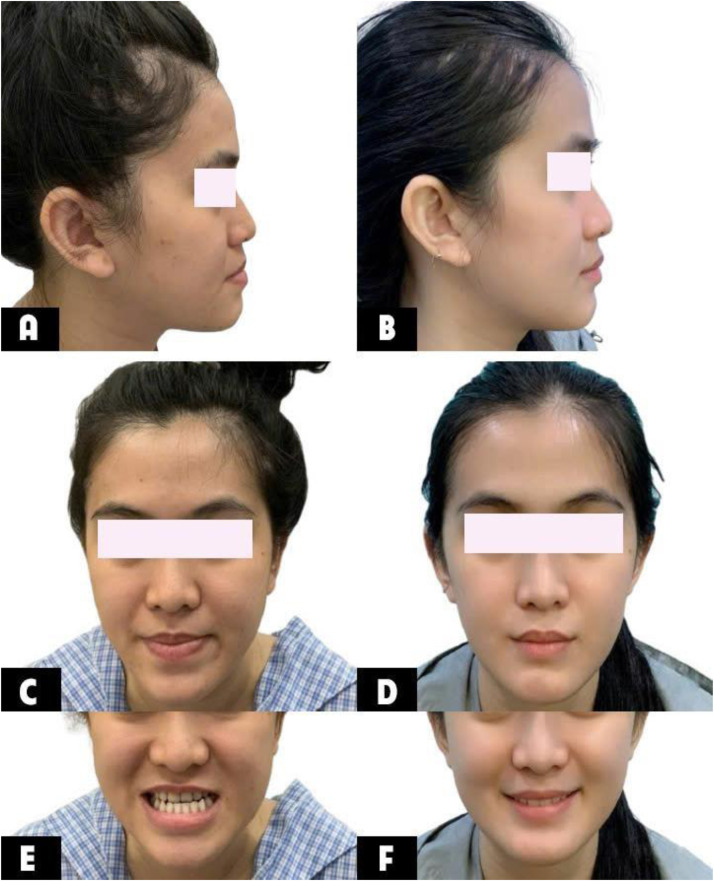
The facial images of a 21-year-old female patient, before (left) and 3 months after BSSRO (SFOA) and mandibular angle reduction (right): A, B – Profile; C, D – Frontal; E, F – Frontal with a slight smile.

**Fig. 4:**
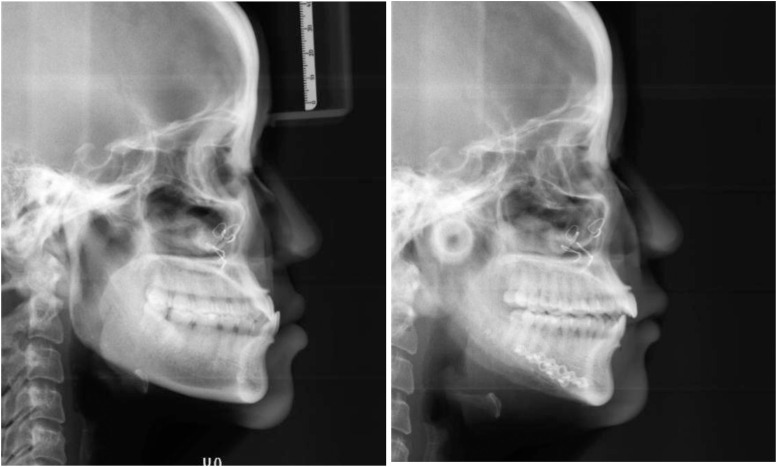
Lateral cephalometric of a 21-year-old female patient before (left) and 3 months after BSSRO (SFOA) and mandibular angle reduction (right).

While this study demonstrated significant differences in early skeletal stability, the results should be interpreted within the context of certain limitations. The primary limitation is the relatively small sample size; however, to validate the reliability of our findings, a post hoc power analysis was conducted based on our primary outcome (horizontal change at Point B), which suggests that the present study retained adequate statistical sensitivity to detect clinically relevant differences between treatment groups. Nevertheless, the small cohort size may have limited our ability to detect statistically significant differences in secondary outcomes with higher variance, such as the horizontal displacement at Pogonion.

A second notable limitation is the relatively short three-month follow-up period. While orthognathic surgery generally requires long-term evaluation (> 1 year) to assess definitive stability, the specific focus of this study was strictly on early skeletal stability. In the Surgery-First Orthognathic Approach (SFOA), the absence of rigid presurgical intercuspation makes the osteotomized segments highly vulnerable to muscle-driven relapse during the immediate postoperative healing phase. Furthermore, the Regional Acceleratory Phenomenon (RAP) is most active during this initial 3 to 4-month window, facilitating rapid bone remodelling and intense orthodontic tooth movement. Therefore, capturing the biomechanical effect of pterygomasseteric sling shortening during this critical early phase is functionally paramount.

Finally, the present study focused strictly on objective cephalometric outcomes. The absence of Patient-Reported Outcome Measures (PROMs) and standardised functional assessments, such as the Index of Orthognathic Functional Treatment Need (IOFTN),^30^^,^^31^ limits our ability to evaluate subjective functional benefits, warranting integration in future prospective protocols. Nonetheless, our research group has initiated a protocol for long-term longitudinal tracking of this cohort to confirm whether this early stabilising effect is maintained beyond the one-year mark.

## Sources of support in the form of grants

None.

## Funding

None.

## Ethical approval

This study was supported by the Independent Ethics Committee IEC, Tra Vinh University (Ref number 185/GCN.ĐC—HĐĐĐ, signed August 25th, 2025); and the IEC of Van Hanh General Hospital (Ref number 01/2025/GCN—HĐĐĐ, signed August 28th, 2025).

## Patient consent

Obtained.

## Declaration of competing interest

None declared.

## References

[1] Rosen H.M. (2017). Evolution of a surgical philosophy in orthognathic surgery. Plast Reconstr Surg.

[2] Minh N.H., Dung T.M., Ngoc V.T.N. (2019). Quality of life and suitability with Vietnamese harmonious face index in class III malocclusion patients. Open Access Maced J Med Sci.

[3] Kim Y., Song S.I., Lee S.H., Sim H.Y., Kim Y.H., Hwa S.C. (2024). Soft tissue paradigm based treatment planning in skeletal class III. Clin Oral Investig.

[4] Seo H.J., Choi Y.K. (2021). Current trends in orthognathic surgery. Arch Craniofacial Surg.

[5] Song I.J., Kang M.S., Lee J.H. (2024). Changes in mandibular width and frontal-lower facial profile after orthognathic surgery using sagittal split ramus osteotomy with removal of internal bone interference in patients with class III skeletal malocclusion. Maxillofac Plast Reconstr Surg.

[6] Chen C.M., Tseng Y.C., Ko E.C., Chen M.Y.C., Chen K.J., Cheng J.H. (2018). Comparisons of jaw line and face line after mandibular setback: intraoral vertical ramus versus sagittal split ramus osteotomies. Biomed Res Int.

[7] Lee C.H., Park H.H., Seo B.M., Lee S.J. (2017). Modern trends in class III orthognathic treatment: a time series analysis. Angle Orthod.

[8] Proffit W.R., Turvey T.A., Phillips C. (2007). The hierarchy of stability and predictability in orthognathic surgery with rigid fixation: an update and extension. Head Face Med.

[9] Kim C.S., Lee S.C., Kyung H.M., Park H.S., Kwon T.G. (2014). Stability of mandibular setback surgery with and without presurgical orthodontics. J Oral Maxillofac Surg.

[10] He X., He J., Yuan H., Chen W., Jiang H., Cheng J. (2019). Surgery-first and orthodontic-first approaches produce similar patterns of condylar displacement and remodeling in patients with skeletal class III malocclusion. J Oral Maxillofac Surg.

[11] Kim G.S., Lim S.H., Jeong S.R., Park J.H. (2021). A surgery-first approach using single-jaw rotational mandibular setback in low-angle mandibular prognathism. Am J Orthod Dentofac Orthop.

[12] Jiang Y., Yang Z., Qi Y. (2023). Early and 1-year postsurgical stability and its factors in patients with complicated skeletal class Ⅲ malocclusion treated by conventional and surgery-first approach: a prospective cohort study. Am J Orthod Dentofac Orthop.

[13] Szegedi L., Rózsa N., Nemes B. (2025). Combined orthodontic, orthognathic surgical, and prosthodontic treatment for severe class III malocclusion using digital workflows. Oper Dent.

[14] Chen C.M., Ting C.C., Cheng J.H., Hsu K.J., Tseng Y.C. (2017). The effect of pterygomasseteric sling’s area in the postoperative stability after mandibular setback surgery. Biomed Res Int.

[15] Kim C.H., Lee J.H., Cho J.Y., Lee J.H., Kim K.W. (2007). Skeletal stability after simultaneous mandibular angle resection and sagittal split ramus osteotomy for correction of mandible prognathism. J Oral Maxillofac Surg.

[16] Chen F., Wu L.P., Terada K., Saito I. (2007). Longitudinal intermaxillary relationships in class III malocclusions with low and high mandibular plane angles. Angle Orthod.

[17] Kuo J.J., Lin C.H., Ko E.W. (2022). Relapse patterns of two-jaw surgical correction in patients with skeletal class III malocclusion and different vertical facial types. Int J Oral Maxillofac Surg.

[18] Barone S., Cevidanes L., Miranda F. (2024). Enhancing skeletal stability and class III correction through active orthodontist engagement in virtual surgical planning: a voxel-based 3-dimensional analysis. Am J Orthod Dentofac Orthop.

[19] Yang X., Piao Z., Liu Y., Chen L., Huang L. (2024). Changes in 3-dimensional measurements of masseter muscle after orthognathic surgery in patients with facial asymmetry. Aesthet Plast Surg.

[20] Ko E.W., Lin S.C., Chen Y.R., Huang C.S. (2013). Skeletal and dental variables related to the stability of orthognathic surgery in skeletal class III malocclusion with a surgery-first approach. J Oral Maxillofac Surg.

[21] Bi D., Gao H., Al-Watary M.Q.H., Sun X., Zhao Q., Li J. (2025). Changes in mandibular angle and intergonial width after bilateral sagittal split ramus osteotomy or bimaxillary surgery with/without counterclockwise rotation. Int J Oral Maxillofac Surg.

[22] Cho H.J. (2007). Long-term stability of surgical mandibular setback. Angle Orthod.

[23] Sun L., Lee K.M. (2019). Three-dimensional evaluation of the postsurgical stability of mandibular setback with the surgery-first approach: comparison between patients with symmetry and asymmetry. J Oral Maxillofac Surg.

[24] Tseng Y.C., Hsiao S.Y., Cheng J.H., Hsu K.J., Chen C.M. (2020). Postoperative skeletal stability and pharyngeal airway: counterclockwise versus clockwise rotation during mandibular setback surgery. Biomed Res Int.

[25] Skariah P.G., Soumithran C. (2025). A prospective study on relapse following bilateral sagittal split osteotomy in mandibular set back approach. J Pharm Bioallied Sci.

[26] Alnasrallah M.M., Toriya T., Kuroe K., Kitahara T., Mori Y., Takahashi I. (2024). Three-dimensional changes in the soft tissue chin after mandibular setback surgery in adult Japanese patients with skeletal class III malocclusion. Clin Investig Orthod.

[27] Haas Junior O.L., Guijarro-Martinez R., de Sousa Gil A.P. (2019). Hierarchy of surgical stability in orthognathic surgery: overview of systematic reviews. Int J Oral Maxillofac Surg.

[28] Troulis M.J., Kearns G.J., Perrott D.H., Kaban L.B. (2000). Extended genioplasty: long-term cephalometric, morphometric and sensory results. Int J Oral Maxillofac Surg.

[29] Jakobsone G., Stenvik A., Sandvik L., Espeland L. (2011). Three-year follow-up of bimaxillary surgery to correct skeletal class III malocclusion: stability and risk factors for relapse. Am J Orthod Dentofac Orthop.

[30] Borzabadi-Farahani A., Olkun H.K., Eslamipour F. (2026). Vertical skeletal patterns as predictors of functional need in orthognathic patients: a retrospective study based on IOFTN scores. J Maxillofac Oral Surg.

[31] Borzabadi-Farahani A. (2025). Systematic review and meta-analysis of the index of orthognathic functional treatment need for detecting subjects with great need for orthognathic surgery. Cleft Palate Craniofac J.

